# Risk factors for ischaemic heart disease in a Cretan rural population: a twelve year follow-up study

**DOI:** 10.1186/1471-2458-7-351

**Published:** 2007-12-18

**Authors:** Ioannis K Karalis, Athanasios K Alegakis, Antonios G Kafatos, Antonios D Koutis, Panos E Vardas, Christos D Lionis

**Affiliations:** 1 Clinic of Social and Family Medicine, School of Medicine, University of Crete, Greece; 2 Department of Cardiology, University Hospital of Crete, Greece; 3 Clinic of Nutrition and Disease Prevention, School of Medicine, University of Crete, Greece

## Abstract

**Background:**

Crete has been of great epidemiological interest ever since the publication of the Seven Countries Study. In 1988 a well-defined area of rural Crete was studied, with only scarce signs of coronary heart disease (CHD) despite the unfavorable risk profile. The same population was re-examined twelve years later aiming to describe the trends of CHD risk factors over time and discuss some key points on the natural course of coronary heart disease in a rural population of Crete.

**Methods and Results:**

We re-examined 200 subjects (80.7% of those still living in the area, 62.4 ± 17.0 years old). The prevalence of risk factors for CHD was high with 65.9% of men and 65.1% of women being hypertensive, 14.3% of men and 16.5% of women being diabetic, 44% of men being active smokers and more than 40% of both sexes having hyperlipidaemia. Accordingly, 77.5% of the population had a calculated Framingham Risk Score (FRS) ≥ 15%, significantly higher compared to baseline (p < 0.001). The overall occurrence rate for CHD events was calculated at 7.1 per 1000 person-years (95% confidence interval: 6.8–7.3).

**Conclusion:**

The study confirms the unfavorable risk factor profile of a well defined rural population in Crete. Its actual effect on the observed incidence of coronary events in Cretans remains yet to be defined.

## Background

The population of Crete has been a subject of great epidemiological interest ever since the Seven Countries Study on cardiovascular diseases reported markedly lower mortality rates for the Cretan cohort, among the 16 initially investigated [1-5]. World Health Organization statistical reports referring to that era are consistent with a lower incidence of coronary heart disease(CHD) in the population [6]. However, we currently have sufficient evidence to support that the natural course of CHD [7] and the cardiovascular risk profile of the population have changed [8-11]. This is mainly attributed to dietary and life-style modifications that have taken place in Crete over the last decades [12-14].

A research project in primary health care was established in 1988, in a rural area of Crete, aiming to identify the cardiovascular risk profile of a pre-defined "low risk" population [15]. The main findings that emerged from this study involved an extremely high prevalence of smoking (particularly among men), hypertension, diabetes and a high occurrence of alcohol intake. Most of the population was found to be obese and presented increased cholesterol levels. Surprisingly, and against this unfavorable risk profile, signs of coronary artery disease were scarce, with only three men fulfilling definite criteria of a previous myocardial infarction [15]. Lacking a certain explanation for this "paradox", the authors suggested a possible cardio-protective role related to the closely-knit social network, the low unemployment rates and the potential benefit of certain dietary habits (e.g. the high consumption of olive oil). Their hypothesis, regarding the presence of a health asset in the community, was further supported by the observation that other Mediterranean countries (Italy, Spain, Portugal, France) sharing many common cultural and dietary characteristics, also ranked at the bottom in the list of cardiovascular morbidity and mortality [16]. Atherosclerosis, however, is a chronic and progressive disease taking decades prior to its clinical presentation [17]. Therefore, the low incidence of ischemic heart disease among men of Crete described in 1988 could simply represent the beneficial inheritance of a healthier way of living in the past. On the other hand, the impact of this emerging unfavorable risk profile, also identified by other investigators [12], would only be adequately assessed 15 to 20 years later.

To highlight these controversies and explore this Cretan "paradox" we decided to carry-out a follow up study involving all inhabitants of the rural area of Spili, in the island of Crete, twelve years after their initial examination, in 1988. The aim of this paper was to describe the trends of CHD risk factors over time and discuss some key points on the natural course of coronary heart disease in this particular rural population of Crete.

## Methods

### Setting and subjects

**Figure 1:**
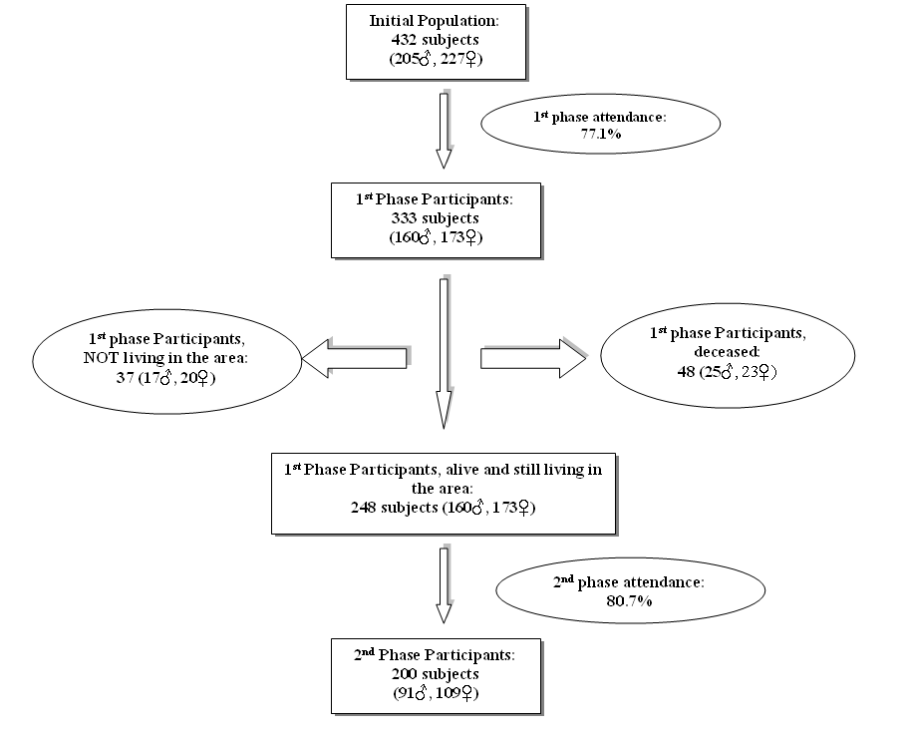
**Study population**. Study population presented schematically. The signs ♂ and ♀ represent men and women respectively.

The study population consisted of all men and women, originally examined in 1988 (n = 333), still living in the rural area of Spili in Crete (Figure 1). The project was launched on September 2000 and was gradually completed over a 15-month period. Thirty-seven subjects of the initial population had meanwhile left the area, while 48 (25 males) had died. Most of the people who had left the village were living either in one of the major cities of the island or in Athens, the reasoning for this internal migration being mainly studies or better professional opportunities. The district nurses and staff of the local Health Centre proved a valuable source of information tracking down each one of the members of the initial population. A total of 248 inhabitants, who participated in the first phase of the study and currently live in the area, represented the target group for the re-examination. They were all given both a formal invitation letter to participate in the study and personally approached by the district nurses. For those deceased, their death certificates and medical records, retrieved from the Health Centre's archive, provided valuable information regarding morbidity and mortality indices of the population. Our study complied with the Declaration of Helsinki while the research protocol was approved by the Ethics Committee of the University Hospital of Crete. All participants provided an informed written consent.

### Medical history and clinical examination

A complete medical history was obtained for each one of the participants. For reasons of comparability to the baseline study, we used the initial questionnaire that was further enriched with topics regarding the subjects' performance status (Boston score, NYHA class) and cardiovascular morbidity over the last decade.

The 10-year Framingham Risk Score (FRS) was calculated for all subjects participating in the first phase of the study, who were free of clinically evident cardiovascular disease (ischemic heart disease, stroke, peripheral vascular disease, heart failure) at baseline examination [18]. The same risk score was re-calculated at follow up (2001), based on the contemporary values of its determinants. Subjects were classified, according to current guidelines [19], into low (<15%), medium (15–25%) and high (>25%) risk categories for developing coronary heart disease (fatal and non-fatal myocardial infarction, unstable angina or angina pectoris) in the following ten years. Data regarding CHD morbidity, defined as non fatal myocardial infarction, unstable angina or angina pectoris, were obtained both through the personal interview during re-examination and through reviewing medical records, kept in the Health Centre's database, for each one of the participants. In most of the cases, a typical medical history of chest pain was associated either with a record of admission to the regional hospital and a discharge diagnosis of coronary artery disease, or a thorough (according to current practice guidelines [20,21]) invasive or non-invasive evaluation, leading to the diagnosis. Both an electronic and a hard copy of patients' records were available at the local Health Centre [15]. For those deceased, the death certificates were obtained and the cause of death was analyzed and classified according to ICD-9 [22]. A CHD death was defined either as a fatal myocardial infarction or as a sudden death with a positive post mortem examination.

### Blood pressure assessment – electrocardiogram

Blood pressure (BP) was assessed for all participants, following a standardized procedure, according to Joint National Committee (JNC) 7 guidelines [23]. Measurements were taken at the end of the interview with the patient seated quietly for at least 5 minutes, feet on the floor and arm supported at heart level. An appropriate sized cuff (with a cuff bladder encircling at least 80% of the arm) was used along with a standard mercury sphygmomanometer. The BP was measured in both arms and in case of a difference the reading from the arm with the higher BP was taken into account. For diastolic BP (DBP) Korotkoff phase V was considered. At least two BP measurements were acquired from each patient with more than 1-min interval among them and the mean value was recorded.

An electrocardiogram (ECG) was obtained for all subjects (Cardiette excel 106 SCP, Elettronica Trentina s.p.s, Cavareno Italy) and was digitally stored in the Practice's database for further analysis.

### Laboratory and somatometric variables

A blood sample was obtained with the patients fasting overnight. Serum was separated and analyzed for glucose, lipids (total cholesterol, HDL, triglycerides), creatinine and γ-Gt. The remaining serum was then frozen at -80°C and kept for subsequent analyses. All analyses were carried out in the local Practice's laboratory (Dry chemistry analyzer Spotchem SP-4410, Menarini, Italy). External quality control for the Practice's lab is routinely carried out twice a year. To further ensure the reliability of our measurements, we conducted an additional external quality control, for a subset of randomly selected specimens, using the laboratory of the University Hospital of Crete as a reference; the standard deviation index (SDI, defined as [lab mean - peer group mean]/peer group standard deviation) was found well below the values of ± 1, as expected.

Weight, height, waist and hip measurements were assessed, as previously described [15]. Body mass index (BMI = weight/height squared, kg/m^2^) and waist to hip ratio were calculated. Smoking habits were recorded through an interview and subjects were classified into non-smokers, ex-smokers (cessation for more than 12 months) and currently active smokers.

### Diagnostic criteria

Hypertension was defined as systolic BP (SBP) ≥ 140 mmHg or diastolic BP (DBP) ≥ 90 mmHg or current treatment with antihypertensive drugs, according to JNC 7 [23]. Awareness of hypertension reflects the subject's knowledge of being hypertensive based on a previous diagnosis. Treatment of hypertension was defined as current use of antihypertensive drugs. Patients who reported a positive or uncertain history of hypertension and had BP below those thresholds without receiving treatment were classified as normotensives. For reasons of comparability, the prevalence of hypertension in 1988 was re-evaluated using current guidelines for its definition.

The diagnosis of diabetes mellitus was based either on a positive medical history, regardless whether the patient was on treatment or not, or on a fasting blood glucose concentration of 126 mg/dL (7.0 mmol/L) or higher, or on a random value of 200 mg/dL (11.1 mmol/L) or higher (confirmed by repeating) and on reporting classic symptoms of hyperglycemia (thirst, polyuria, weight loss, visual blurring) [24]. Again, the current diagnostic criteria were used to re-assess the prevalence of diabetes in the 1988 data.

National Cholesterol Education Program guidelines – NCEP III (Adult Treatment Panel [ATP] III) were used to assess the prevalence of hypercholesterolemia in the population [19]. For patients without known medical history of cardiovascular disease, a total cholesterol value ≥ 200 mg/dl was considered as reason for intervention (usually dietary).

Overweight patients were defined as those with a BMI value ≥ 25 kg/m^2^, while the criterion for obesity and extreme obesity was ≥ 30 and ≥ 40 kg/m^2 ^respectively [25-27]. The prevalence of abdominal obesity was assessed by measuring waist circumference; according to current practice guidelines a value greater than 102 cm for men and 88 cm for women was considered indicative of central type obesity [25].

### Statistical analysis

Statistical analyses were performed by a statistician (A.A.) using the Statistical Package for Social Science (SPSS version 13.0) for Windows. Continuous variables were expressed as mean ± standard deviation (SD) while discrete ones as counts and proportions. Paired samples t-test was used for two group's comparisons. Associations between categorical variables were measured using Pearson's chi-square statistic (Mantel-Haesnzel for ordinal), while McNemar's or McNemar-Bowker χ^2^-statistic was assessed to estimate changes in proportions between phase 1 and phase 2 variables such as smoking hyperlipidaemia, etc.

Survival time (in months) was expressed as mean ± standard deviation (measured in months, presented as Kaplan-Meier survival curves). Differences in survival times were assessed by Mantel Cox statistic.

## Results

As schematically shown in Figure 1, a total number of 200 patients (mean age ± SD: 62.4 ± 17.0 years old, 91 males) were re-examined, the overall attendance rate for those still living in the area of Spili reaching 80.7% (Table 1). In order for our data to be comparable to the initial study, results are presented with the population divided into the same age groups used in 1988 (≤ 44, 45–64, 65–79) [15] with the addition of a fourth one (≥80), due to aging (Table 2). Forty-eight subjects (27 males and 21 females) refused our invitation since "they felt in good health" and they had very demanding working activities restricting their free time. This subgroup of "non-attendees" did not differ significantly in terms of age (χ^2 ^= 1.435, df = 2, p = 0.488), sex (χ^2 ^= 1.794, df = 1, p = 0.180) or their baseline calculated FRS (p = 0.120) compared to those re-examined. Another 37 subjects (17 males, most of them in the younger age group) had meanwhile left the area of Spili, this representing an access barrier.

**Table 1:** Population analysis based on participation in second phase

Participation	Age groups (1988)					
			≤ 44	45–64	65–79	Total

Participants in phase 2	Sex	Male	36 (39.6%)	44 (48.4%)	11 (12.1%)	91
		Female	40 (36.7%)	43 (39.4%)	26 (23.9%)	109
	Total		76	87	37	200
Deceased	Sex	Male	3 (12.0%)	6 (24.0%)	16 (64.0%)	25
		Female	0 (0.0%)	8 (34.8%)	15 (65.2%)	23
	Total		3	14	31	48
Moved from the area	Sex	Male	14 (82.4%)	1 (5.9%)	2 (11.8%)	17
		Female	14 (70.0%)	2 (10.0%)	4 (20.0%)	20
	Total		28	3	6	37
Not participants	Sex	Male	15 (55.6%)	10 (37.0%)	2 (7.4%)	27
		Female	7 (33.3%)	10 (47.6%)	4 (19.0%)	21
	Total		22	20	6	48

Attendance for the Spili cohort in phase 2, according to their sex and age group. Participation has been calculated according to the age group the subjects belonged at baseline evaluation.

**Table 2:** Common determinants for cardiovascular disease in the population (mean values ± SD)

**MALES**								
**Age groups (phase 2)**	**SBP (mmHg)**	**DBP (mmHg)**	**Glu (mg/dl)**	**Chol (mg/dl)**	**Tg (mg/dl)**	**HDL (mg/dl)**	**BMI (kg/m**^2^**)**	**Waist (cm)**

**≤ 44 years **(n = 18)	128.3 ± 12.5	86.6 ± 9.8	80.6 ± 9.1	178.4 ± 33.1	96.6 ± 30.0	42.3 ± 13.7	26.8 ± 4.0	96.3 ± 12.0
**45–64 years **(n = 28)	148.8 ± 21.7	95.6 ± 13.3	94.8 ± 44.1	193.8 ± 34.9	131.8 ± 68.5	44.0 ± 10.4	26.3 ± 3.1	97.0 ± 8.2
**65–79 years **(n = 37)	151.6 ± 23.5	88.5 ± 13.5	108.0 ± 43.3	204.3 ± 44.5	112.8 ± 64.3	49.7 ± 13.8	26.8 ± 4.1	100.0 ± 11.1
**≥ 80 years **(n = 8)	158.8 ± 23.4	86.9 ± 13.6	105.8 ± 18.8	231.3 ± 51.0	150.1 ± 95.5	42.4 ± 11.9	26.4 ± 3.5	96.3 ± 6.2
**Total **(n = 91)	146.8 ± 22.9	90.2 ± 13.1	98.3 ± 38.6	198.3 ± 42.0	118.7 ± 64.6	45.8 ± 12.9	26.6 ± 3.7	98 ± 10.1

**FEMALES**								

**≤ 44 years **(n = 16)	118.0 ± 10.7	78.3 ± 7.7	80.9 ± 8.6	172.8 ± 30.5	74.4 ± 36.0	51.1 ± 11.3	23.2 ± 2.0	78.8 ± 7.0
**45–64 years **(n = 33)	135.2 ± 21.8	84.9 ± 11.2	86.9 ± 20.4	197.2 ± 34.6	103.6 ± 30.3	47.4 ± 14.5	29.3 ± 3.8	94.0 ± 11.4
**65–79 years **(n = 31)	153.4 ± 21.2	86.6 ± 9.8	112.5 ± 73.3	207.7 ± 34.3	121.9 ± 72.0	51.5 ± 14.8	30.8 ± 4.7	97.7 ± 11.4
**≥ 80 years **(n = 23)	160.5 ± 23.9	82.5 ± 12.9	105.8 ± 36.8	195.9 ± 38.4	135.9 ± 59.6	52.5 ± 18.7	28.3 ± 4.4	98.2 ± 11.4
**Total **(n = 109)	144.2 ± 25.2	84.0 ± 10.9	98.7 ± 48.6	196.9 ± 36.1	112.4 ± 57.5	50.4 ± 15.1	28.7 ± 4.7	93.9 ± 12.6

The number of subjects for each group is displayed in parenthesis. These values refer to data collected in 2001. SBP: Systolic Blood Pressure, DBP: Diastolic Blood Pressure, Glu: Glucose, Chol: Cholesterol, Tg: Triglycerides

The average values of the most common determinants for cardiovascular disease are presented in Table 2. One should note the particularly high levels of systolic blood pressure (146.8 ± 22.9 for men and 144.2 ± 25.2 mmHg for women, respectively), fasting blood glucose (98.3 ± 38.6 and 98.7 ± 48.6 mg/dl) and cholesterol (198.3 ± 42.0 and 196.9 ± 36.1 mg/dl, respectively). Obesity, as expressed with the BMI and waist circumference values represents another issue with the average values for our population being well above normal limits, particularly for the subset of women exceeding the age of 65 (30.8 ± 4.7 kg/m^2 ^for those aged 65–79 years).

Comparing the recently obtained data to the baseline characteristics of our population in 1988, presented in Table 3, we observe an increase in the prevalence of hypertension in almost every age group examined, for both sexes (60.5% of men – 52 subjects and 65.1% of women – 71 subjects). A similar unfavorable tendency is observed for diabetes, particularly in women, while obesity represents these days a more important problem compared to 1988. Smoking habits have almost remained unchanged with the exception of middle aged women (45–64 years, not shown in the table) where it has significantly increased (1 case in 1988 compared to 7 cases in 2001, p < 0.001). Cholesterol levels have decreased compared to baseline in most of the age groups of our population, for both sexes (p < 0.05 for both men and women).

**Table 3:** Prevalence of most common risk factors, for both sexes, in 1988 and 2001

	**Males**			**Females**		
	**2001**	**1988**	**p**	**2001**	**1988**	**p**
	
**Hypertension (%)**	52 (60.5%)	33 (20.6%)	<0.001	71 (65.1%)	71 (41.0%)	<0.001
**Diabetes (%)**	10 (12.0%)	11 (6.9%)	0.065	11 (12.8%)	15 (8.7%)	<0.001
**Hypercholesterolemia (%)**	36 (43.4%)	101 (63.1%)	<0.001	37 (43.0%)	105 (60.7%)	<0.001
**Increased w.c (♂ > 102 cm – ♀ > 88 cm, %)**	27 (32.5%)	25 (15.6%)	<0.001	50 (58.1%)	83 (48.0%)	<0.001
**Current smokers (%)**	37 (44.6%)	73 (45.6%)	0.454	12 (14.0%)	18 (10.4%)	1.000

w.c: waist circumference, ♂: males, ♀: females

**Figure 2:**
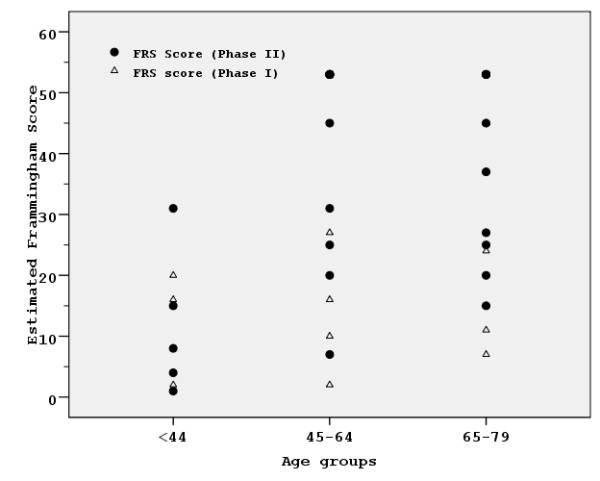
**FRS in 1988 and 2001**. Framingham Risk Score (FRS) scatter plots in comparable age groups at baseline (1988, Phase I) and follow up (2001, Phase II).

The described risk profile modification and the population gradual aging, lead to a significant increase of the calculated Framingham Risk Score (t = 15.764, df = 196, p < 0.001), as shown in Figure 2. Moreover, as reported in Table 4, a 25.5% of those characterized as "low-risk" in 1988 (10-year estimated absolute risk for developing coronary heart disease less than 15%) and 78.6% of those in "medium-risk" category (15–25%) were classified in 2001 as "high-risk" (>25%) regarding the likelihood for future coronary events. A reverse risk profile modification was not observed since merely 4 subjects characterized in 1988 as "medium or high-risk" were classified in 2001 in a lower risk category (McNemar-Bowker test χ^2 ^= 104.790 df = 3, p < 0.001).

**Table 4:** Framingham Risk Score modifications through time (1988 – 2001)

		**FRAMINGHAM RISK SCORE 2001**			
			
		**<15%**	**15–25%**	**>25%**	**Total**
**FRAMINGHAM RISK SCORE 1988**	**<15%**	62 (42.8%)	46† 31.7%)	37† (25.5%)	145 (100%)
	**15–25%**	2‡ (4.8%)	7 (16.7%)	33† (78.6%)	42 (100%)
	**>25%**	0 (0.0%)	2‡ (20.0%)	8 (80.0%)	10 (100%)
			
	**Total**	64 (32.5%)	55 (27.9%)	78 (39.6%)	197 (100%)

Distribution of the participants in the three FRS groups and modifications through time. We can see that only 4 subjects (‡) moved to a lower FRS group in 2001 compared to baseline, while the vast majority (†) has worsen its profile.

**Figure 3:**
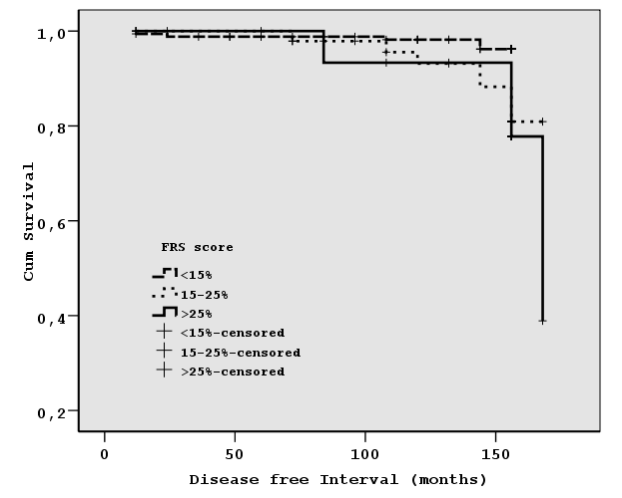
**Survival curves according to the FRS group**. Coronary Heart Disease survival free curves for each one of the FRS groups. Test of equality of survival distributions for different FRS groups: Log Rank (Mantel-Cox) χ^2 ^= 13.828, df = 2, p < 0.001.

Using death certificates, Practice's medical registry and information personally obtained during the interviews, we identified a total number of 21 events indicative of CHD in our population (14 cases of myocardial infarction or CHD death and 7 cases of unstable angina and angina pectoris), over the follow up period of 12 years. A survival curve was plotted for the disease-free intervals of each one of the FRS groups discussed (Figure 3). Our data indicate a definite dissociation among the curves, since the 6^th ^year of follow up, with more CHD events occurring in the medium and high risk groups (log-rank χ^2 ^= 13.828, df = 2, p < 0.001). Isolating the subset of patients in the highest risk group (FRS > 25% in 1988), we identified 10 subjects (representing 46% of the total number of 22) who remain still alive at the end of the follow up period in 2001, 7 of them with no apparent signs of cardiovascular disease. Overall, five subjects (22%) of the high risk group developed CHD throughout the follow up period, while twelve subjects (54%) had no signs of cardiovascular disease and were either still alive or had died of a different cause.

The overall occurrence rate of CHD events was calculated at 7.1 per 1000 person-years (95% CI: 6.8–7.3), while the corresponding values for the three FRS groups were 2.9 per 1000 person-years (95% CI: 2.8–3.1) for the low, 15.2 per 1000 person-years (95% CI: 14.0–16.3) for the medium and 18.9 per 1000 person-years (95% CI: 16.7–21.2) for the high risk group respectively.

## Discussion

This follow up study underlines that the unfavorable risk profile identified in 1988 [15], for most of the common determinants of cardiovascular disease, is still present. However, signs of clinically evident CHD were scarce, both at baseline and at follow up evaluation. To the best of our knowledge there are very limited data in the literature concerning epidemiological studies, where a geographically well-defined population is monitored over time without the application of any large scale intervention.

Multiple epidemiological studies, some of them based on population samples of the Seven Countries study, provide evidence regarding the unfavorable risk profile modification of the Greek population and an increasing tendency in CHD prevalence [10,28,29]. In the area of Spili, the unfavorable risk profile not only remains, but seems to get even worse, with most of the indices exceeding those of the urban (and also younger) cohort in the ATTICA study [30]. Smoking habit is very frequent among men (more than 40% of the male population are active smokers) while it has become more common in women. Hypertension, diabetes and dyslipidaemia are present in alarming rates and the same is the case for obesity, which has significantly increased, probably as a result of the sedentary life style (following the shift from farming to other forms of occupational activity). The increasing prevalence of hypertension or metabolic disorders can likewise be considered a 'natural' epiphenomenon of the aging process [31]. The statement is doubted however by other studies, like the one performed among a Tanzanian, Brazilian and Italian population, where blood pressure was correlated with age only in the latter two cohorts whereas in the African population who consumed a low salt "fish and vegetable" diet, blood pressure did not increase with age [32]. The only favorable risk factor modification we report is a slight cholesterol level decrease compared to baseline, a common finding to other epidemiological studies in European populations, mainly attributed to dietary changes [33,34].

Modifications regarding the prevalence of most common risk factors are quantified and easier perceived with the calculation of FRS for both time points (1988 and 2001) in our population. Only a minority seems to remain in the same risk group category or reduce its chances for future coronary events while the vast majority has moved into higher risk groups, confirming evidence from previous epidemiological studies carried out in Cretan cohorts [10,28]. Considering the fundamental effect of aging in FRS calculation, this would not be unexpected twelve years past the initial evaluation. However, FRS is estimated to be much higher in 2001, compared to 1988, even when similar age groups are compared (Figure 2).

FRS seems to overestimate the risk for coronary events in European populations and certain literature reports argue against its use [35-37]. In our study, it has successfully identified subjects with the highest risk for development of coronary events. The survival curves plotted in Figure 3, indicating periods free of fatal or non-fatal coronary events, demonstrate a clear dissociation, early during the follow up period, among the three predefined risk groups. Certain methodological issues in some of the aforementioned studies [36] can partly explain the reduced incidence of CHD events recorded; the absence of an electrocardiogram screening and therefore the inability to identify cases of silent ischemia (rather common particularly in the elderly women and diabetics) is one of them. In certain cases, an obscure medical history may lead to failure to classify certain sudden deaths related to coronary heart disease as such. This was not the case in our study, where the population was closely monitored thanks to an efficient 'primary care surveillance' program, with all valuable information examined (prior to registration) in terms of reliability and 'cross checked' plausibility.

Over the 12 years of follow up and for the 248 subjects monitored (200 who were re-examined and 48 who died and whose medical records and death certificates were examined) we identified only 21 coronary events, 10 of them fatal. Despite the fact that the small numbers (of both the sample size and events) do not allow us to draw safe conclusions, the calculated occurrence rate of 7.1 (95% CI: 6.8–7.3) CHD events per 1000 patient-years seems rather low considering the aforementioned risk profile and the advanced age of our population sample. Previous reports have also demonstrated an impressive survival for the Cretan cohort compared to others of the Seven Countries Study, despite the increased prevalence of risk factors for cardiovascular disease [38]. An adequate explanation however, is lacking.

## Limitations

A major limitation of this study has to do with the 85 subjects who were initially examined in 1988 and were not re-assessed in 2001. Almost half of them (48 subjects) refused our invitation while 37 could not be accessed since they were not living in the area anymore. Our statistical analysis reveals that, based on baseline characteristics, those who refused our invitation were not statistically different in terms of age, sex or calculated FRS compared to participants. We can therefore assume that having those subjects re-examined, our results wouldn't be significantly different. On the other hand, those who have meanwhile left the area, mostly belonging to the younger age groups, would only be expected to show limited incidence ranking of coronary artery disease. We therefore have reasons to believe that even if those patients had been approached, the reported CHD morbidity and mortality would have only been (if at all) favorably affected.

Another limitation of the present study is that there were only single measurements of relevant risk factors that can be rather misleading particularly for the diagnosis of hypertension. When this study was initiated, this specific matter constituted an issue of major speculation. The difficulty was overcome with the thorough use of Spili Practice medical records where multiple readings were usually recorded for each one of our patients. When a definite and solid conclusion could not be drawn, a re-evaluation visit was scheduled within a two-month period.

## Conclusion

The study confirms the unfavorable risk factor profile of a well defined rural population in Crete. However, the initial question that has inspired this project since 1988 remains illusive. The assumption that closely knit social networks may be associated with the good health status Cretans enjoy [15] remains still unanswered. Arguments referring to the presence of a protective factor (or condition) against the development of CHD in the population have to be further supported. Other parameters, determining the health asset of a population, like dietary habits (including the consumption of Cretan herbs among residents of the Spili area [39]) and the influence of the traditional Cretan social network were not included in this study. An ongoing study that monitors the Spili cohort and associates CHD rates with psychological scales, measuring stress management, could further explore the links between different coronary morbidity patterns and specific socio-cultural influences.

## List of abbreviations

CHD: Coronary Heart Disease

FRS: Framingham Risk Score

NYHA: New York Heart Association

NCEP: National Cholesterol Education Program

ATP: Adult Treatment Panel

BP: Blood Pressure

SBP: Systolic Blood Pressure

DBP: Diastolic Blood Pressure

JNC: Joint National Committee

ECG: Electrocardiogram

SDI: Standard Deviation Index

BMI: Body Mass Index

SPSS: Statistical Package for Social Science

SD: Standard Deviation

CI: Confidence Interval

## Competing interests

The author(s) declare that they have no competing interests.

## Authors' contributions

IK carried out the research project in the rural area of Spili, examining all participants and reviewing their medical records kept in the local Practice. He has also drafted the manuscript. AA performed the statistical analysis and critically reviewed the drafts. AGK has critically reviewed and modified the drafts of the manuscript. ADK, who performed the first phase of the study 12 years earlier, helped in the design of the current project and in the evaluation of our results. PV helped in the design of the project while CL conceived the research project, supervised both the first and second phase, participated in data collection and helped draft the manuscript. All authors read and approved the final manuscript.

## Pre-publication history

The pre-publication history for this paper can be accessed here:


